# Urban-rural disparities in wife-beating attitude among married women: a decomposition analysis from the 2017 Senegal Continuous Demographic and Health Survey

**DOI:** 10.1186/s13690-021-00612-5

**Published:** 2021-06-15

**Authors:** Betregiorgis Zegeye, Gebretsadik Shibre, Bright Opoku Ahinkorah, Mpho Keetile, Sanni Yaya

**Affiliations:** 1 HaSET Maternal and Child Health Research Program, Shewarobit Field Office, Shewarobit, Ethiopia; 2 Department of Reproductive, Family and Population Health, School of Public Health, Addis Ababa University, Addis Ababa, Ethiopia; 3 School of Public Health, Faculty of Health, University of Technology Sydney, Ultimo, NSW 2007 Australia; 4 Population Studies and Demography, University of Botswana, Gaborone, Botswana; 5 School of International Development and Global Studies, University of Ottawa, 120 University Private, Ottawa, ON K1N 6N5 Canada; 6 The George Institute for Global Health, Imperial College London, London, UK

**Keywords:** Women, Domestic violence, Attitudes, Autonomy, Global Health, Senegal

## Abstract

**Background:**

Globally, intimate partner violence is one of the most common forms of gender-based violence, and wife beating is one component of intimate partner violence, with the problem being more severe among women living in rural settings. Little is known about the factors that explain the urban-rural disparity in the prevalence of wife beating attitude in Senegal. In this paper, we aimed to decompose the urban-rural disparities in factors associated with wife beating attitude among married women in Senegal.

**Methods:**

Data were derived from the 2017 Senegal Continuous Demographic and Health Survey. We used the Blinder-Oaxaca decomposition method to decompose and explain the variation in the prevalence of disagreement to wife beating between urban and rural areas in Senegal.

**Results:**

The results show that 48.9% of married women in Senegal disagreed with wife-beating. About 69% of urban women disagreed with wife beating, but only 36% of rural women disagreed with wife beating. About 68.7% of women in the sample reported that they disagreed to wife beating by their husbands for burning food and nearly 50% of women reported that they disagreed with wife beating when they refuse to have sex with their husbands. About 86% of the urban-rural disparities in disagreement with wife beating are explained in this study. Economic status (45.2%), subnational region (22.4%), women’s educational status (13.3%), and husband’s educational status (10.7%) accounted for 91.6% of the disparities.

**Conclusions:**

The study shows urban-rural disparities in the prevalence of wife-beating attitude (disagreement with wife beating) and this disfavored rural residents. We suggest the need for the government of Senegal to consider pro-rural equity strategies to narrow down the observed disparities. Moreover, socioeconomic empowerment and attitudinal changing interventions using existing socio-cultural institutions as platforms can be used to deliver such interventions.

## Background

Gender-based violence (GBV) is any type of gender-related violence against women, leading to the suffering of women and resulting in physical, psychological and sexual abuse, either in public or private life [1]. Globally, more than one-third of ever-married or cohabiting women have experienced some form of GBV either through sexual or physical violence from their intimate partner [2]. The problems are very common in African countries [3, 4], where life time prevalence of physical and/or sexual intimate partner violence among ever-partnered women is as high as 36.6% [4]. Despite laws against domestic violence in Senegal since 1999, many women still do not have access to information about GBV. Consequently, the practice continues to be highly accepted since the legal bodies such as the judiciary and the police do not properly enforce laws against GBV [5]. Due to unsatisfied response and care from the government officials and even at health institutions, many victimized women often do not seek treatment and become reluctant to report any case of GBV such as wife-beating [2, 5, 6].

In many low-and middle-income countries, there is a general acceptance of ‘wife beating’–a common type of intimate partner violence (IPV)–often perpetuated by the commonly held norms and gender roles in the society [7]. For instance, it is generally believed that a man has the right to assert power over a woman and correct any deviant female behavior [7, 8], using physically punitive measures such as beating [9]. A woman’s attitude towards wife-beating is considered a proxy for her perception of her status [10, 11]. The perception that IPV is supported and culturally normative are amongst the utmost major factors related with the possibility of enactment and social replies to perpetration [12–15]. Women who consider that wife beating is acceptable and normative are likely to allow themselves to be violated, and to develop lifelong psychological problems, and most commonly keep it a secret instead of reporting to legal bodies and their families or close friends [16]. More than the criminal or victim, the societal attitude towards wife beating could highly govern the reply and correction of the behavior. In societies where IPV is culturally accepted and normative, supporting victims and the response to wife beating behavior by the community members is highly unlikely [17, 18]. A woman who considers such violence as not acceptable is likely to be aware of her greater sense of worth, self-esteem, status, and to reflect positively on her sense of empowerment [16–19]. On the other hand, a woman who considers such violent behavior as ‘justifiable’, accepts the right of her husband to control her behavior even by means of violence [10, 20]. Evidence shows that the magnitude of IPV is often higher among women who justify wife beating by their husbands and perceive it as a healthy life [21–24].

Several studies in sub-Saharan-Africa [25], Ethiopia [26], Egypt [27], and Nigeria [23], have shown that non-acceptance of wife beating varies among urban and rural residents. In Senegal, domestic violence including wife beating is very common [5, 28, 29]. For instance, the percentage of women who believe that a woman’s beating by her husband for any of the reasons such as when a wife argues with him; refuses to have sex; burns the food; goes out without telling him; or when she neglects the children decreased from 65.2% in 2005 to 45.7% in 2017. However, the figure indicates that the problem still needs attention and proper intervention [30].

Although wife beating is a socially acceptable practice in Senegal, especially in rural settings [5], there is a dearth of evidence regarding the magnitude and factors associated with disparities in wife beating attitude. As a result, the study aimed at decomposing the rural-urban disparities in factors associated with wife beating attitude (disagreement with wife beating) among married women in Senegal using the nationally representative data from the 2017 Senegal Continuous Demographic and Health Survey (SCDHS).

## Methods

### Study area

Located in West Africa, Senegal is well-known as the “Entry to Africa”. Up to half of its 15.4 million people (2016) live in and around Dakar and other urban areas [31, 32]. Senegal has a long history of carrying out Demographic and Health Surveys (DHS). Before the start of the SCDHS, and Continuous Service Provision Assessment [CSPA]), Senegal had six rounds of DHSs conducted in 1986, 1992–93, 1997, 1999, 2005, and 2010–11; and two Malaria Indicator Survey (MISs) conducted in 2006 and 2008–09 [31]. All these surveys were nationally representative surveys of women of reproductive age and collected information on fertility and reproductive health.

### Data sources and sampling procedure

We used the most recent and available 2017 SCDHS for this analysis [32]. All variables included in the dataset were checked and recoded to ensure standardized response categories in the survey. This survey is publicly available on the DHS website (https://dhsprogram.com/methodology/survey/survey-display-534.cfm). The 2017 SCS samples were selected using a stratified, two-stage cluster sampling design to provide estimates for the health and demographic variables of interest for the country. Large geographic settings known as enumeration areas (EA) were selected in the first stage through Probability Proportional to Size (PPS). Household listing was completed in each EA to ready the sampling frame. A fixed number of households were randomly selected from each EA in the second stage. The survey included 8380 households, about 78,950 and 74,985 un-weighted and weighted household members, respectively. Selected participants were questioned using standard and country-specific questions covering a wide range of health topics. A total of 11,394 married women (15–49 years of age) were included for analysis. The details of the survey methodology are outlined in the 2017 SDHS Final Report [32]. We used the individual recode file (IR) for the analysis.

### Study variables

#### Outcome variable

Women’s attitude towards wife beating by their husbands was the outcome variable of the study. In the DHS, data on this variable was collected to allow measurements of women’s empowerment. Currently married women at the time of the survey administration answered five questions which have been used to indirectly assess whether they disagree that a husband is justified in beating his wife. The questions asked whether the husband is justified to beat his wife if she: a) burns food b) argues with him c) goes out without telling him d) neglects the children and e) refuse to have sexual intercourse with him. According to the DHS guideline, a woman is said to be empowered if she disagreed to all of these reasons. Based on this, an overall binary variable was created with a value of 1 and 0, where 1 indicated disagreement to all of the reasons, and 0 indicated agreement with at least one of the conditions for wife beating [33].

#### Equity stratifier

Place of residence (urban versus rural) was the equity stratifier of which disparities in wife beating attitude (disagreement to wife beating) were examined.

#### Confounders

The correlates of attitude towards wife-beating were selected based on previous studies [7, 12, 15, 19, 25, 26, 34–37]. The selected correlates included: age of women, employment for cash, religion, women’s educational level, husband's educational level, husband's occupation, wealth index, subnational region, media exposure and decision-making. Media exposure was created based on whether an individual was exposed to one or more of the following media at least once a week: newspaper or magazine, radio, television vs. not. Decision making variable was created based on three questions that were asked of the women:1) person who usually makes decisions on health care for yourself, 2) person who usually makes decisions on making major household purchases, and 3) person who usually makes decisions on visits to your family or relatives. Each of these three areas of decision making was coded into a binary variable with a value of 1 and 0, where 1 indicated that the woman decided alone, or together with her husband, and 0 if she did not participate in the above three decision making parameters. We finally created an overall variable that reflects whether a woman participated in the aforementioned three decision-making areas. The overall variable had three categories: 0 (no empowerment), 1–2 (moderate empowerment) and 3 (high empowerment). We followed a similar strategy that was used in a previous study [38]. The categories of each of the correlates have been described in Tables 1 and 2.

**Table 1:** Frequency distribution of participants and urban-rural proportions across confounding variables, Evidence from 2017 Senegal Continuous-DHS

Variables	Frequency (%)	Place of residence (%)		Difference (%) (Rural-Urban)
		Urban	Urban	
**Women’s age**				
15–19	1045 (9.17)	10.42 (9.41–11.52)	4.46 (3.61–5.49)	5.96 (5.80, 6.03)
20–24	1906 (16.73)	17.99 (16.96–19.06)	12.27 (11.15–13.49)	5.72 (4.69–5.57)
25–29	2237 (19.63)	19.19 (18.18–20.25)	19.95 (18.53–21.44)	0.76 (− 0.35, − 1.19)
30–34	2200 (19.31)	19.10 (18.04–20.21)	21.08 (19.40–22.86)	−1.98 (− 1.36, − 2.65)
35–39	1678 (14.73)	14.18 (13.11–15.33)	16.69 (15.03–18.49)	−2.51 (− 1.92, −3.16)
40–44	1379 (12.10)	11.80 (10.93–12.73)	14.40 (13.17–15.72)	− 2.6 (− 2.24, − 2.99)
45–49	949 (8.33)	7.28 (6.68–7.93)	11.13 (9.70–12.74)	−3.85 (− 3.02, − 4.81)
**Women’s educational level**				
No educated	7294 (64.02)	73.92 (71.88–75.85)	40.71 (37.87–43.63)	33.21 (34.01, 32.22)
Primary school	2407 (21.13)	16.74 (15.30–18.29)	31.69 (29.46–34.01)	−14.95 (− 14.16, − 15.72)
Secondary school	1493 (13.10)	8.79 (7.83–9.84)	21.46 (19.63–23.40)	−12.67 (− 11.80, − 13.56)
Higher	199 (1.75)	0.53 (0.37–0.77)	6.12 (4.68–7.97)	−5.59 (− 4.31, − 7.20)
**Religion**				
Muslim	11,097 (97.39)	98.10 (97.22–98.70)	96.12 (94.50–97.28)	1.98 (2.72, 1.42)
Others	297 (2.61)	1.89 (1.29–2.77)	3.87 (2.71–5.49)	−1.98 (− 1.42, − 2.72)
**Media exposure**				
No	1097 (9.63)	10.86 (9.42–12.50)	1.69 (1.31–2.17)	9.17 (8.11, 10.33)
Yes	10,297 (90.37)	89.13 (87.49–90.57)	98.30 (97.82–98.68)	−9.17 (−10.33, −8.11)
**Wealth index**				
Poorest	3101 (27.22)	34.07 (30.37–37.99)	2.20 (1.54–3.12)	31.87 (28.83, 34.87)
Poor	2706 (23.75)	31.42 (28.43–34.57)	4.62 (3.47–6.11)	26.8 (24.96, 28.46)
Middle	2569 (22.55)	21.74 (18.64–25.20)	18.55 (16.23–21.12)	3.19 (2.41,4.08)
Rich	1762 (15.46)	8.37 (6.48–10.76)	33.78 (30.02–37.75)	−25.41 (−23.54, −26.99)
Richest	1256 (11.02)	4.37 (2.71–6.95)	40.83 (36.50–45.31)	−36.46 (−33.79, −38.36)
**Employment for cash**				
No cash-based employment	1550 (24.77)	31.13 (27.24–35.32)	5.08 (4.13–6.23)	26.05 (23.11, 29.09)
Cash-based employment	4707 (75.23)	68.86 (64.67–72.75)	94.91 (93.76–95.86)	−26.05 (− 29.09, − 23.11)
**Decision making**				
No empowerment	7318 (64.23)	65.39 (62.94–67.76)	43.10 (39.68–46.58)	22.29 (21.18, 23.26)
Moderate empowerment	2763 (24.25)	24.20 (22.18–26.35)	37.54 (34.90–40.26)	−13.34 (− 12.72, − 13.91)
High empowerment	1313 (11.52)	10.39 (9.06–11.89)	19.35 (17.23–21.66)	−8.96 (− 8.17, −9.77)
**Region**				
Dakar	830 (7.28)	2.23 (1.82–2.73)	49.36 (45.89–52.83)	−47.13 (−44.07, −50.10)
Ziguinchor	491 (4.31)	2.90 (2.04–4.11)	2.86 (2.38–3.42)	0.04 (−0.34, 0.69)
Diourbel	976 (8.57)	19.10 (17.46–20.85)	4.32 (3.43–5.44)	14.78 (14.03, 15.41)
Saint-Louis	771 (6.77)	6.01 (5.11–7.07)	7.09 (6.39–7.85)	−1.08 (−0.78, 1.28)
Tambacounda	879 (7.71)	7.96 (6.77–9.35)	2.52 (2.07–3.08)	5.44 (4.70, 6.27)
Kaolack	733 (6.43)	7.67 (6.57–8.94)	5.42 (4.65–6.32)	2.25 (1.92, 2.62)
Thies	930 (8.16)	12.01 (10.33–13.92)	14.05 (12.13–16.21)	−2.04 (−1.80, −2.29)
Louga	881 (7.73)	9.58 (8.08–11.32)	3.25 (2.63–4.02)	6.33 (5.45, 7.30)
Fatick	850 (7.46)	7.01 (6.16–7.97)	2.26 (1.86–2.73)	4.75 (4.30, 5.24)
Kolda	870 (7.64)	7.05 (6.12–8.12)	3.10 (2.57–3.74)	3.95 (3.55, 4.38)
Matam	864 (7.58)	5.37 (4.43–6.48)	1.93 (1.51–2.48)	3.44 (2.92, 4.00)
Kaffrine	989 (8.68)	7.32 (6.46–8.28)	1.93 (1.52–2.46)	5.39 (4.94, 5.82)
Kedougou	589 (5.17)	1.42 (1.01–2.00)	0.76 (0.60–0.97)	0.66 (0.41, 1.03)
Sedhiou	741 (6.50)	4.29 (3.71–4.97)	1.07 (0.84–1.37)	3.22 (2.87, 3.60)
**Husband's occupation**				
Didn’t work	419 (3.68)	3.09 (2.56–3.72)	4.36 (3.47–5.46)	−1.27 (−0.91, −1.74)
Professional or technical or managerial	1218 (10.69)	6.40 (5.51–7.43)	20.10 (17.67–22.78)	−13.7 (− 12.16, − 15.35)
Sales	1942 (17.04)	18.31 (16.64–20.10)	19.08 (16.96–21.38)	− 0.77 (− 0.32, − 1.28)
Agricultural-self-employed	2738 (24.03)	31.17 (28.24–34.25)	4.14 (3.28–5.21)	27.07 (24.96, 29.04)
Skilled manual	1627 (14.28)	12.22 (10.64–14.00)	17.85 (16.04–19.82)	− 5.63 (− 5.40, − 5.82)
Unskilled manual	1735 (15.23)	14.48 (13.13–15.95)	20.30 (18.35–22.40)	−5.82 (− 5.22, − 6.45)
Others	1715 (15.05)	14.30 (12.72–16.04)	14.14 (12.64–15.78)	0.16 (0.08, 0.26)
**Husband's educational level**				
No educated	8363 (73.40)	82.55 (80.40–84.51)	51.10 (48.05–54.15)	31.45 (32.35, 30.36)
Primary school	1382 (12.13)	9.16 (8.00–10.47)	19.93 (18.20–21.79)	−10.77 (− 10.2, − 11.32)
Secondary school	1158 (10.16)	6.32 (5.35–7.44)	18.83 (16.89–20.94)	−12.51 (− 11.54, − 13.50)
Higher	491 (4.31)	1.95 (1.42–2.67)	10.11 (8.16–12.47)	−8.16 (−6.74, −9.80)
**Burning food**				
Agree/accept/justify	3563 (31.27)	33.68 (31.57–35.86)	15.67 (13.84–17.69)	18.01 (17.73, 18.17)
Disagree/not accept/not justify	7831 (68.73)	66.31 (64.13–68.42)	84.32 (82.30–86.15)	−18.01 (− 18.17, − 17.73)
**Neglecting children**				
Agree/accept/justify	5329 (46.77)	50.27 (47.78–52.76)	26.64 (24.30–29.11)	23.63 (23.48, 23.65)
Disagree/not accept/not justify	6065 (53.23)	49.72 (47.23–52.21)	73.35 (70.88–75.69)	−23.63 (− 23.65, − 23.48)
**Arguing with husband**				
Agree/accept/justify	5541 (48.63)	52.60 (50.11–55.08)	25.35 (23.13–27.70)	27.25 (26.98, 27.38)
Disagree/not accept/not justify	5853 (51.37)	47.39 (44.91–49.88)	74.64 (72.29–76.86)	−27.25 (− 27.38,-26.98)
**Refuse for sex**				
Agree/accept/justify	5736 (50.34)	55.59 (53.09–58.07)	25.15 (22.86–27.59)	30.44 (30.23, 30.48)
Disagree/not accept/not justify	5658 (49.66)	44.40 (41.92–46.90)	74.84 (72.40–77.13)	−30.44 (− 30.48, − 30.23)
**Goes out without telling husband**				
Agree/accept/justify	5272 (46.27)	50.94 (48.56–53.30)	23.05 (20.90–25.36)	27.89 (27.66, 27.94)
Disagree/not accept/not justify	6122 (53.73)	49.05 (46.69–51.43)	76.94 (74.63–79.09)	−27.89 (− 27.94, − 27.66)
**Wife beating Attitude**				
Agree/accept/justify	6684 (51.1)	62.61 (60.23–64.93)	34.70 (31.96–37.54)	27.91 (27.39, 28.27)
Disagree/not accept/not justify	4710 (48.9)	37.38 (35.06–39.76)	65.29 (62.45–68.03)	−27.91 (− 27.39, − 28.27)
**Place of residence**				
Urban	4292 (37.67)	NA	NA	NA
Rural	7102 (62.33)	NA	NA	NA

*NA* not applicable

**Table 2:** Wife-beating attitude across explanatory variables: Evidence from 2017 Senegal Continuous-DHS

Variables	Wife-beating		***P***-value
	Agreed N**o** (%)	Disagreed N**o** (%)	
**Women’s age**			*P* < 0.001
15–19	717 (68.61)	328 (31.39)	
20–24	1185 (62.17)	721 (37.83)	
25–29	1277 (57.09)	960 (42.91)	
30–34	1276(58.00)	924 (42.00)	
35–39	972 (57.93)	706 (42.07)	
40–44	752 (54.53)	627 (45.47)	
45–49	505 (53.21)	444 (46.79)	
**Women’s educational level**			*P* < 0.001
No educated	4857 (66.59)	2437 (33.41)	
Primary school	1191 (49.48)	1216 (50.52)	
Secondary school	617 (41.33)	876 (58.67)	
Higher	18 (9.05)	181 (90.95)	
**Place of residence**			*P* < 0.001
Urban	1875 (43.69)	2417 (56.31)	
Rural	4809 (67.71)	2293 (32.29)	
**Religion**			*P* < 0.001
Muslim	6572 (59.22)	4525 (40.78)	
Others	112 (37.71)	185 (62.29)	
**Media exposure**			*P* < 0.001
No	872 (79.49)	225 (20.51)	
Yes	5812 (56.44)	4485 (43.56)	
**Wealth index**			*P* < 0.001
Poorest	2463 (79.43)	638 (20.57)	
Poor	1796 (66.37)	910 (33.63)	
Middle	1376 (53.56)	1193 (46.44)	
Rich	716 (40.64)	1046 (59.36)	
Richest	333 (26.51)	923 (73.49)	
**Employment for cash**			*P* < 0.001
No cash-based employment	1022 (67.37)	495 (32.63)	
Cash-based employment	2535 (54.82)	2089 (45.18)	
**Decision making**			*P* < 0.001
No empowerment	4701 (64.24)	2617 (35.76)	
Moderate empowerment	1386 (50.16)	1377 (49.84)	
High empowerment	597 (45.47)	716 (54.53)	
**Region**			*P* < 0.001
Dakar	195 (23.49)	635 (76.51)	
Ziguinchor	180 (36.66)	311 (63.34)	
Diourbel	516 (52.87)	460 (47.13)	
Saint-Louis	362 (46.95)	409 (53.05)	
Tambacounda	687 (78.16)	192 (21.84)	
Kaolack	341 (46.52)	392 (53.48)	
Thies	429 (46.13)	501 (53.87)	
Louga	507 (57.55)	374 (42.45)	
Fatick	519 (61.06)	331 (38.94)	
Kolda	696 (80.00)	174 (20.00)	
Matam	519 (60.07)	345 (39.93)	
Kaffrine	813 (82.20)	176 (17.80)	
Kedougou	480 (81.49)	109 (18.51)	
Sedhiou	440 (59.38)	301 (40.62)	
**Husband's occupation**			*P* < 0.001
Didn’t work	222 (52.98)	197 (47.02)	
Professional or technical or managerial	446 (36.62)	772 (63.38)	
Sales	1122 (57.78)	820 (42.22)	
Agricultural-self-employed	2046 (74.73)	692 (25.27)	
Skilled manual	881 (54.15)	746 (45.85)	
Unskilled manual	958 (55.22)	777 (44.78)	
Others	1009 (58.83)	706 (41.17)	
**Husband's educational level**			*P* < 0.001
No educated	5457 (65.25)	2906 (34.75)	
Primary school	655 (47.40)	727 (52.60)	
Secondary school	449 (38.77)	709 (61.23)	
Higher	123 (25.05)	368 (74.95)	

#### Statistical analysis

The analysis for this paper was conducted as follows. First, descriptive analysis such as frequency distribution of the outcome variable, stratifier (place of residence) and confounders were conducted. Within this, the distribution of the outcome variables across the equity stratifier (place of residence) and confounders was presented using frequency distribution tables and bar graphs. Second, Bivariate analysis (using Pearson chi-square test) was conducted to select candidate confounder variables that affected disagreement with wife beating, using *p*-value less than 0.05 cut point. Then, multicolliniarity test was carried out using variance inflation factor (VIF) to check whether or not there was collinearity among selected explanatory variables and there was confirmation that no evidence of multicolliniarity existed (VIF Mean = 1.38, VIF Min = 1.01, VIF Max = 2.39).

Finally, we decomposed the urban-rural disparity in the prevalence of disagreement with wife beating using the Blinder-Oaxaca (BO) decomposition method [39]. The BO decomposition method has been commonly applied in the labor market area to decompose the mean log wages between male and female, and between other subgroups such as race [39, 40]. The procedure divides the gap in the mean wage between the groups of interest and attributes the gap in the mean wage into explained and unexplained portions. The explained portion of the decomposition is attributed to the difference in the distribution of characteristics and variables of between the groups, and the unexplained part is treated as discrimination and or difference between the groups in unobservable characteristics.

The BO decomposition can be applied in other fields such health, where disparity in health or health care indicators can be analyzed and decomposed between two groups such as between poor and rich. In our study, the two groups are urban and rural settings, and the measure of health indicators is attitude towards wife beating. We ran a logistic regression-based decomposition analysis to see how prevalence of wife beating varies by place of residence. In the regression analysis, the category of the outcome variable of interest was disagreement with wife beating. We used the *Oaxaca stata* module to do the decomposition. While the module is basically meant for linear models, it can also be used for the probit and logit models as well [40]. We took the weight, cluster and strata design elements into account during the analysis. All analyses were carried out in Stata version.14 for windows.

#### Ethical clearance

We did our analysis using data that is publicly available. Since, the dataset is already available in the public domain, no ethical approval was required for this study. Details about data and ethical standards are available at: http://goo.gl/ny8T6X.

## Results

### Socio-demographic characteristics of respondents

A total of 11,394 currently married women participated in the survey. From this total, 19.6% were in the ages 25–29 years and 64% had no formal education. About 62.3% of the participants were rural residents and 97.4% were Muslims. Nearly one-quarter (24.8%) of the respondents had no cash-based employment. Regarding media exposure, 90.4% of the participants indicated that they read a newspaper, listened to a radio or watched television less than once a week or at least once a week. Concerning decision making, 64.2% of the married women had no decision-making power. Only 11.5% of the respondents had decided either alone or together with their husbands in all of the three decision making parameters (about her health, to make household purchases and to visit relatives/families) (Table 1).

The number of currently married women who justified wife beating at least for one of the five reasons was 6684 (51.1%).

**Fig. 1:**
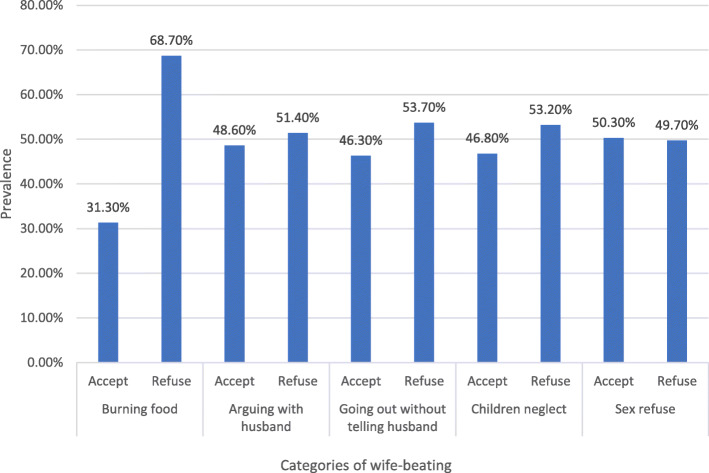
Percentage distribution of wife-beating attitude among currently married women by reasons for wife beating in Senegal: Evidence from 2017 Senegal Continuous Survey

More than half (50.3%) of the married women accepted wife beating if the women refused sex. Similarly, the proportion of married women who accepted wife beating for arguing with the husband and going out without telling the husband were approximately 48.6 and 46.3% respectively (Fig. 1).

### Prevalence of wife-beating across explanatory variables

Wife-beating attitude varied based on women’s age, with a higher proportion of older women disagreeing with wife beating. For instance, about 31.4% of women within the 15–19 years age group disagreed with wife-beating, while the prevalence increased to 46.8% among women within the 45–49 years age groups. Wife-beating attitude significantly varied based on women’s educational status. For instance, about 91% of women who had attended higher education disagreed with wife beating. However, only 33.4% of women with no formal education disagreed with wife-beating. The results also showed nearly 53% difference in disagreement with wife-beating between poorest women (20.6%) and richest women (73.5%) (Table 2).

### Association between place of residence and wife beating attitude

As shown in Table 3, we found that women and husband’s educational level, and region were factors associated with wife beating attitude (disagreement with wife beating) for both urban and rural residents. However, wealth index and media exposure were factors associated with wife beating attitude among women living in rural areas, but not for urban residents.

**Table 3:** Association between place of residence and wife beating attitude (disagreement with wife beating) among currently married women: Evidence from 2017 Senegal C-DHS

Variables	Rural		Urban	
	Coefficient [95% CI]	***P***-value	Coefficient [95% CI]	***P***-value
**Women’s age**				
15–19	Ref		Ref	
20–24	−0.33 (− 0.81, 0.14)	0.169	− 0.36 (−1.20, 0.47)	0.392
25–29	− 0.21 (− 0.67, 0.24)	0.366	− 0.15 (− 0.92, 0.61)	0.694
30–34	− 0.29 (− 0.70, 0.11)	0.160	0.05 (− 0.76, 0.88)	0.893
35–39	0.02 (− 0.40, 0.45)	0.918	− 0.18 (− 1.01, 0.63)	0.653
40–44	0.11 (− 0.31, 0.55)	0.586	0.04 (− 0.76, 0.85)	0.915
45–49	−0.06 (− 0.57, 0.45)	0.812	0.08 (− 0.67, 0.85)	0.821
**Women’s educational level**				
No educated	Ref		Ref	
Primary school	0.42 (0.18, 0.66) **	0.001	0.43 (0.17, 0.68) **	0.001
Secondary school	0.26 (−0.14, 0.67)	0.206	0.55 (0.21, 0.89) **	0.001
Higher	1.47 (0.02, 2.91) *	0.046	1.57 (0.59, 2.54) **	0.002
**Husband education**				
No educated	Ref		Ref	
Primary school	0.12 (−0.21, 0.46)	0.486	0.50 (0.15, 0.86) **	0.005
Secondary school	0.29 (−0.10, 0.69)	0.153	0.41 (0.03, 0.79) *	0.034
Higher	0.97 (0.08, 1.86)*	0.032	0.60 (0.06, 1.14) *	0.029
**Employment for cash**				
No cash-based employment	Ref		Ref	
Cash-based employment	−0.22 (− 0.47, 0.02)	0.080	− 0.07 (− 0.57, 0.42)	0.770
**Husband occupation**				
Didn’t work	Ref		Ref	
Professional or technical or managerial	−0.48 (−1.11, 0.14)	0.131	0.24 (− 0.34, 0.83)	0.415
Sales	−0.40 (− 0.88, 0.06)	0.091	0.17 (− 0.41, 0.77)	0.557
Agricultural-self-employed	−0.39 (− 0.84, 0.04)	0.077	− 0.18 (− 0.80, 0.43)	0.562
Skilled manual	− 0.41 (− 0.87, 0.04)	0.078	0.14 (− 0.41, 0.70)	0.610
Unskilled manual	− 0.30 (− 0.78, 0.17)	0.215	0.20 (− 0.36, 0.78)	0.473
Others	− 0.33 (− 0.81, 0.14)	0.175	− 0.15 (− 0.83, 0.52)	0.652
**Wealth index**				
Poorest	Ref		Ref	
Poor	0.02 (−0.22, 0.26)	0.868	0.07 (−0.63, 0.78)	0.832
Middle	0.26 (−0.05, 0.58)	0.099	0.17 (−0.42, 0.77)	0.559
Rich	0.53 (0.10, 0.95) *	0.015	0.39 (−0.22, 1.01)	0.209
Richest	0.79 (−0.01, 1.59)	0.053	0.59 (−0.07, 1.27)	0.081
**Media exposure**				
No	Ref		Ref	
Yes	0.53 (0.19, 0.87) **	0.002	−0.41 (− 0.93, 0.11)	0.122
**Religion**				
Muslim				
Others	0.32 (−0.31, 0.95)	0.318	−0.18 (−1.00, 0.62)	0.650
**Region**				
Dakar	Ref		Ref	
Ziguinchor	−1.10 (− 3.07, 0.87)	0.272	−0.25 (− 0.71, 0.20)	0.281
Diourbel	−1.54 (− 3.58, 0.48)	0.134	− 0.86 (− 1.64, − 0.08) *	0.029
Saint-Louis	− 1.71 (− 3.76, 0.33)	0.101	− 0.52 (− 0.92, − 0.13) **	0.009
Tambacounda	−3.06 (− 5.13, − 0.99) **	0.004	−0.77 (− 1.31, − 0.23) **	0.005
Kaolack	−2.13 (− 4.16, − 0.10) *	0.039	−0.04 (− 0.60, 0.52)	0.886
Thies	− 1.65 (− 3.64, 0.33)	0.103	− 0.52 (− 0.94, − 0.09) *	0.017
Louga	− 1.76 (− 3.77, 0.25)	0.087	− 0.94 (− 1.34, − 0.55) ***	0.000
Fatick	− 2.38 (− 4.38, − 0.37) *	0.020	−0.74 (− 1.47, − 0.02) *	0.043
Kolda	− 3.57 (− 5.62, − 1.53) **	0.001	−1.09 (− 1.67, − 0.52) ***	0.000
Matam	−2.08 (− 4.09, − 0.06) *	0.043	−0.45 (− 1.03, 0.12)	0.123
Kaffrine	− 2.99 (− 5.05, − 0.93) **	0.005	−1.68 (− 2.45, − 0.92)***	0.000
Kedougou	−3.30 (− 5.36, − 1.23)**	0.002	−1.58 (− 2.09, − 1.07) ***	0.000
Sedhiou	− 1.88 (− 3.88, 0.11)	0.065	− 0.33 (− 1.38, 0.71)	0.532
**Decision making**				
No empowerment	Ref		Ref	
Moderate empowerment	0.10 (− 0.10, 0.32)	0.322	0.13 (− 0.16, 0.42)	0.375
High empowerment	0.21 (− 0.17, 0.61)	0.276	0.27 (− 0.09, 0.64)	0.145

*Ref* Reference, *CI* Confidence Interval

* significant at *p* < 0.05; ** significant at *p* < 0.01; *** significant at *p* < 0.001

### Factors associated with urban-rural disparities in wife-beating attitude

**Fig. 2:**
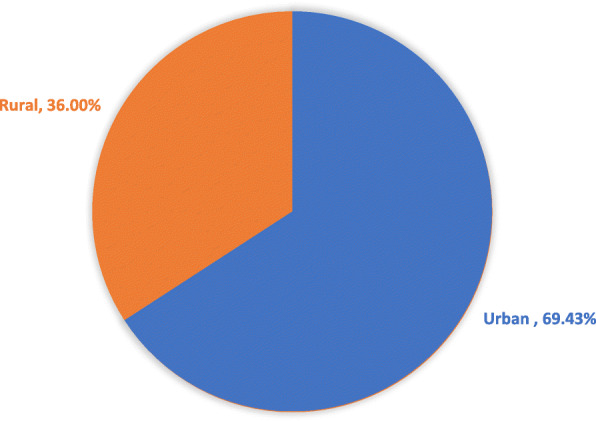
Urban-rural disparities in disagreed with wife-beating among currently married women in Senegal: Evidence from 2017 Senegal Continuous Survey

A number of factors were observed to have significant associations with wife-beating attitude such as decision making, employment for cash, maternal education, husband’s education, husband’s occupation, economic status (wealth index), subnational region, religion, maternal age, and media exposure. The current study shows that the percentage of wife-beating attitude among urban married women was 69.4% (95% CI: 0.66, 0.72) as shown in Table 3. Whereas among the rural residents, it was 36% (95% CI: 0.33, 0.39) as shown in Table 4. About 33.4% of the disparities between the two subgroups in disagreement with wife-beating were observed in Senegal in 2017 (Fig. 2).

**Table 4:** Factors that explained urban-rural disparities in disagreement with wife-beating in Senegal using Blinder-Oaxaca decomposition method: Evidence from 2017 Senegal C-DHS

Wife-beating attitude (Disagreed with wife beating)	Coefficients (95% CI)	***P***. Value
Urban	0.69 (0.66, 0.72)	< 0.001
Rural	0.36 (0.33, 0.39)	< 0.001
Difference	0.33 (0.29, 0.37)	< 0.001
Explained	0.29 (0.24, 0.33)	< 0.001
Unexplained	0.05 (−0.02, 0.11)	0.140
**Factors that explained urban-rural disparities for disagreed with wife-beating**		
Employment for cash	−0.01 (− 0.02, 0.01)	0.105
Media exposure	0.01 (0.003, 0.014)	0.003
Women education	0.04 (0.02, 0.05)	< 0.001
Wealth quintile	0.13 (0.09, 0.17)	< 0.001
Decision making	0.02 (0.01, 0.03)	0.001
Religion	0.001 (−0.001, 0.003)	0.416
Husband occupation	−0.002 (− 0.008, 0.004)	0.607
Women’s age	0.01 (0.003, 0.013)	0.001
Region	0.07 (0.05, 0.08)	< 0.001
Partner educational level	0.03 (0.02, 0.05)	< 0.001

Wealth index (45.2%), subnational region (22.4%), women’s educational status (13.3%), and husband’s educational status (10.7%) accounted for 91.6% of the disparities.

## Discussion

The current study sought to decompose the rural-urban disparities in factors associated with wife beating attitude among married women in Senegal. To the best of our knowledge, this is the first attempt to decompose urban-rural disparities in disagreement with wife beating among married women using Blinder-Oaxaca technique. In this study, the percentage of married women who believed wife beating is justifiable was 51.1%. This finding calls for the need to eliminate domestic violence among women, families and communities in general [1]. Significant differences were observed in disagreement with wife-beating between urban and rural women with 33.4% higher among the urban residents as compared to their rural counterparts. Comparable with prior studies in sub-Saharan Africa [25], Ethiopia [26], Egypt [27], Nigeria [23] and elsewhere [19], this study also showed that women living in rural settings were less likely to disagree with wife beating as compared to their urban counterparts. The plausible reason for this could be the impact of commonly prevalent traditional beliefs, norms and values that spread and continued for several decades across rural settings, while virtually lessening in urban places because of the rapid globalization and modernization effect [35]. The deep rooted and customary culture in Africa which makes gender disparities a source of pride for men with control over women in decision-making explains the observed attitudes towards wife-beating [16, 19, 37]. This socially accepted behaviour that views men as superior and women as subordinates worsen the susceptibility of women to IPV or wife beating as also shown by some studies in Zambia [41–43]. Concerning exposure to legal information, in Senegal, the percentage of women accessing legal services continue to be low, particularly in rural and peri-urban areas, because of the economic, social and cultural barriers that exist [5].

Household economic status was a major contributor for wife beating attitude, and accounted for 45.2% of the disparities. Several previous studies reported that wife beating could be significantly affected by economic status [25, 26, 34, 36]. Scholars suggest that accepting wife beating by poor women might be due to their reliance on their husbands for living [44]. If one person’s livelihood is totally dependent on another individual, there is the likelihood of acceptance of actions by the independent person, even if negative [45]. On the contrary, due to the possibility of getting access to some resources even if the marriage is going to dissolve, women living in wealthier households have the tendency to disagree with wife-beating [27]. Women with low socioeconomic status are more likely to have been exposed to the practice during their childhood, compared to women in better socio-economic status who had better access and exposure to media, consequently having knowledge about their rights and recognizing the globally applicable gender equity norm [36]. In Senegal, despite the nation’s law and family Code 1972 granting and giving equal rights to women and men to become decision makers, owner of land and other resources, the persistence of socio-cultural barriers and customs prevent them from being treated equally in practice. Based on the custom, women cannot accede to land, rather it supports and guarantees power to husbands [5].

Subnational region accounted for 22.6% of urban-rural disparities in disagreement with wife beating among married women in Senegal. As supported by previous studies [19, 36], variations in wife beating across subnational regions, might be due to regional norms and attitude, as well as economic and social structures [37]. Moreover, heterogeneous tendencies across regions may be attributed to variability in ethnic or cultural norms and socioeconomic differences [23]. Similarly, other scholars also suggested that variation in attitudes towards wife beating across regions is the reflection of the diverse sociocultural settings in the country [26, 46]. Since wife beating is a manifestation of the social, cultural and behavioral transformation of a given society in its evolution towards a more gender egalitarian society [26], we believe the effect of region are because of socio-cultural differences in women’s status and decision-making empowerment within their jurisdiction.

Maternal educational status accounted for 13.4% for urban-rural disparities in wife beating attitude. This is supported by a prior study elsewhere [19]. Women who at least attended primary school were less likely to accept that wife beating is justifiable as compared to women who had no formal education [16, 25]. Since education is the key means of gaining knowledge and increasing decision making freedom and capacity, education has great effect on disagreement with wife beating.

Women in higher educational status are less dependent on their husbands, and this can help the women to disagree with wife beating [47]. Moreover, wife beating is considered as a way of sharing household resources to show supremacy and the full control of resources [48]. As a result, besides being the key indicator of the women’s status in the community, education is also another best intervention to end gender-based violence in the society [47]. The positive relationship between education and women’s resistance of wife beating signifies that empowering women educationally can hugely help in ensuring gender equality and reduction of adverse physical, mental and sexual and reproductive health consequence from gender-based violence as well [35] that perpetuate masculinity in traditional societies like Senegal.

Husband’s education accounted for 10.8% of urban-rural disparities in wife beating attitude. Our study also revealed that the level of husband’s education was one of the key determinants of wife beating attitude as shown by previous studies [34]. It is also plausible, that an educated husband can be democratic and solve problems through discussion compared to a non-educated husband [49]. Decision making power of the women was another explanatory factor accounting for 5.25% urban-rural disparities in disagreement with wife beating among married women in Senegal. Evidence suggests that as an indicator of empowerment, women’s decision-making has one of the strongest positive associations across multiple developmental outcomes [50]. Decision making can greatly help in understanding the range to which women can control and participate in handling resources, manage household resources and their rights [51].

Women’s age and media exposure explained nearly 6% of the disparities, consistent with previous studies [26, 35]. Younger women, especially in the new marriage unions, may accept wife beating since they are new in the environment and culture and are not able to resist [52]. Regarding media exposure, previous research has found that such awareness can influence a wide range of attitudes and behaviors [53]. Access to media information is expected to have inverse relationship with justification of beating a wife, because of dissimilarities in awareness about human rights, law and other ways of protection of rights (23, [23]. Evidence shows that low media exposure leads women to support wife beating than their counterparts who have much access to media information [16, 25].

The main strength of the study is the use of a nationally representative data, to identify the explanatory variables to explain the urban-rural disparities in wife-beating attitude in Senegal. Identifying factors and explaining disparities in wife-beating attitudes can be used to guide interventions used to narrow down the disparities and empower women in the country. However, the study should be seen with the following limitations. First, the proportion of contribution of each category of the explanatory variables is not well-known. Second, due to the cross-sectional nature of the study, causal-effect relationship was not possible to ascertain.

## Conclusions

The findings highlight that household economic status, subnational region, women’s and husband’s educational level, women’s decision-making power, women’s age, and media exposure were the main explanatory variables responsible for the urban-rural differences in wife beating attitude among married women in Senegal. Policy makers need to focus on designing interventions that are geared towards boosting women’s and their husband’s socioeconomic status so as to increase women’s outlook in disagreement with wife beating practices. Again, giving more attention to women residing in regions with low prevalence of disagreement with wife-beating may be needed. Finally, improving quality of family relationship to change attitudinal predisposition to wife beating among couples using existing socio-cultural institutions as platforms to deliver such interventions should be considered.

## Data Availability

Data for this study were sourced from Demographic and Health surveys (DHS) and available here: http://dhsprogram.com/data/available-datasets.cfm.

## Abbreviations

DHS: Demographic and Health Survey
EA: Enumeration Areas
GBV: Gender Based Violence
SCS: Senegal Continuous Survey
